# A Case of Chronic Thromboembolic Pulmonary Hypertension

**DOI:** 10.4021/cr187w

**Published:** 2012-05-20

**Authors:** Xin Jin, Sang-Hoon Seol, Bo-Min Park, Jae-Kyun Kim, Tae-Jin Kim, Pil-Sang Song, Dong-Kie Kim, Ki-Hun Kim, Doo-Il Kim

**Affiliations:** a Division of Cardiology, Department of Internal Medicine, Yanbian Second People's Hospital, Yanbian, China; b Division of Cardiology, Department of Internal Medicine, Inje University College of Medicine, Haeundae Paik hospital, Busan, Korea

**Keywords:** Chronic thromboembolic pulmonary hypertension, Transthoracic echocardiography, Pulmonary embolism

## Abstract

Chronic thromboembolic pulmonary hypertension (CTEPH) is a severe complication of incomplete resolution of large pulmonary embolism (PE).Transthoracic echocardiography (TTE) and chest computed tomography (CT) are useful for the diagnosis and follow-up of CTEPH. We report a case of 40-year-old male who wasadmitted with gradually aggravated dyspnea in recent 2 years and had history of acute PE 10 years ago, was detected CTEPH by TTE and confirmed with chest CT.

## Introduction

Chronic thromboembolic pulmonary hypertension (CTEPH) is a form of pulmonary hypertension caused by obstruction and vascular remodeling of pulmonary arteries [1]. It is observed in 2-4% of patients after acute pulmonaryembolism (PE) and related with significant morbidity and mortality [2, 3]. We report a case of CTEPH following a history of acute PE and briefly review the diagnosis and treatment of CTEPH.

## Case Report

**Figure 1:**
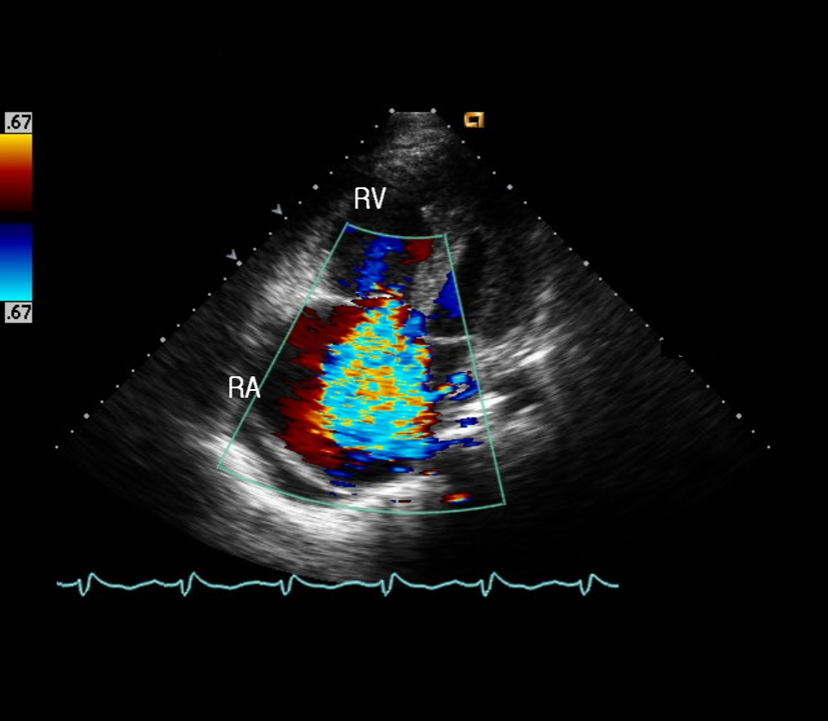
Transthoracic echocardiography at the apical 4 chamber view showed right ventricular and atrial dilatation and severe tricuspid valvular regurgitation. RA, right atrium; RV, right ventricle.

**Figure 2:**
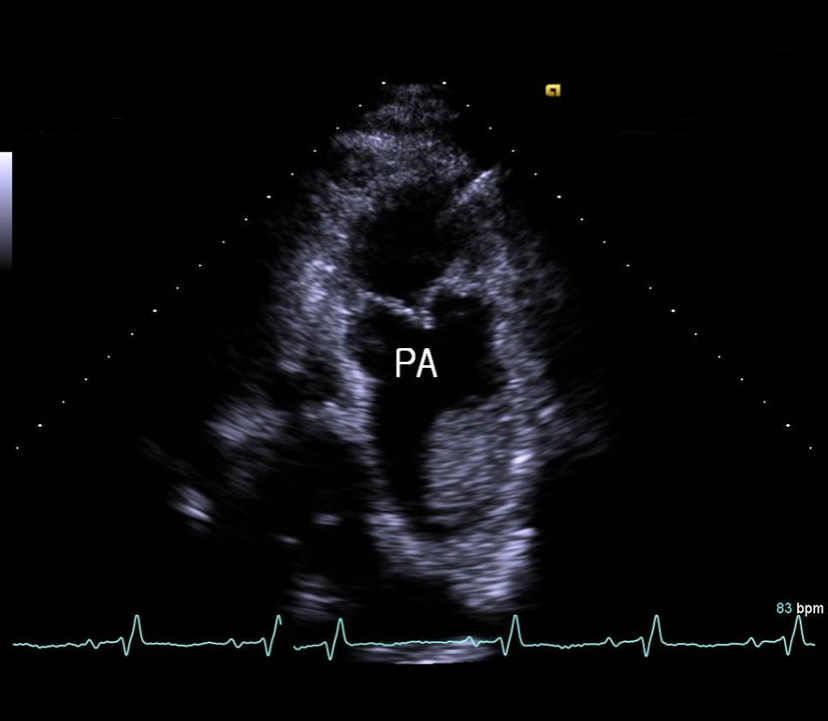
Transthoracic echocardiography at the modified parasternal short axis view showed proximal pulmonary artery thrombus. PA, pulmonary artery.

**Figure 3:**
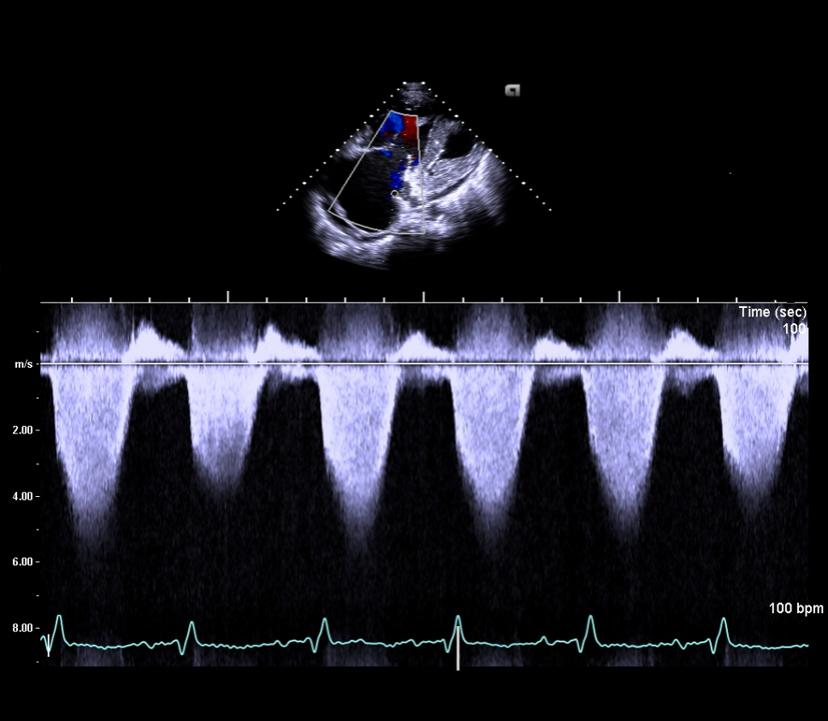
Continuous wave doppler signal showed the peak tricuspid valvular regurgitation velocity was 4.2 m/sec and peak pressure gradient was estimated at 70.56 mmHg.

**Figure 4:**
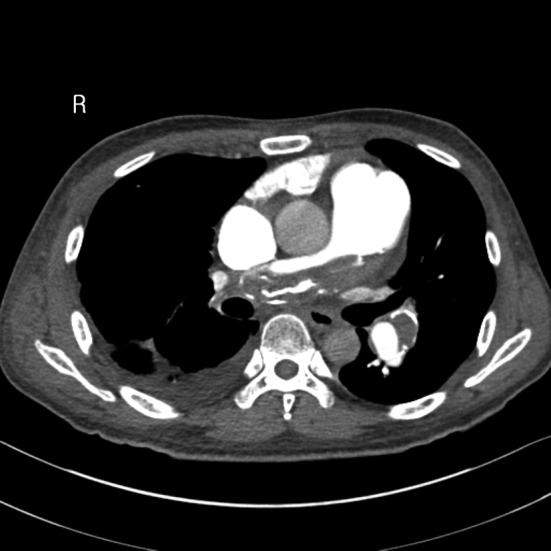
Chest computed tomography showed diffused thrombus in the central and both main pulmonary arteries.

A 40-year-old male wasa truck driver without family history and admitted with progressively worsening shortness of breath from 2 years ago. He had history of acute PE 10 years ago and treated with anticoagulant therapy whichwas stopped by himself after 6 months.Physical examination revealed blood pressure 100/60 mmHg, pulse rate 110 beats/minute, respiration rate 24 breaths/minute, jugular venous distention, and pitting edema in both lower extremities. Blood tests revealed D-dimer 6.04 µg/mL, pro-BNP 6963 pg/mL, and platelet 97×10^9^/L. Artery blood gas analysis (room air) revealed pH 7.47, PCO_2_ 23 mmHg, PO_2_ 67 mmHg, and O_2_saturation 92%. In addition, factor V Leiden was negative and protein C and S levels, anticardiolipin antibodies and lupus anticoagulant were within normal limits. Electrocardiogram showed sinus tachycardia, right axis deviation, and right ventricle hypertrophy. Chest X-ray revealed enlargement of the right ventricle and both main pulmonary arteries, as well as bilateral pleural effusion. Initial transthoracic echocardiography (TTE) showed enlarged right chambers with right overload and severe tricuspid regurgitation (Fig. 1), and a massive thromboembolus (4.35×2.85 cm) in the enlarged pulmonary artery (Fig. 2) with severe pulmonary hypertension(Fig. 3). Chest computed tomography (CT) revealed eccentric filling defect in the central and both main pulmonary arteries (Fig. 4). CT venogram showed diffuse thrombus from the suprarenal to the femoral vein. Therefore, CTEPH was diagnosed. The patient has been considered pulmonary thromboendarterectomy but he refused to undergo surgery and received anticoagulant therapy of heparin for 3 weeks. Then follow-up inferior vena cava venogram revealed that the thrombus had disappearedand the patient’s symptom mild improved.He was discharged with warfarin and prostacyclin analogue therapy. 3 months later,follow-upTTE and chest CT showed there was no significant improvement.

## Discussion

CTEPH is defined as mean pulmonary artery pressure greater than 25 mmHg that persists 6 months after PE is diagnosed [4].It is observed in 2-4% of patients after acute PE [2, 3].Numerous risk factors predispose patients to develop CTEPH, including acute PE, splenectomy, infection, abnormal expression of procoagulant proteins and so on [1].CTEPH is related to the increased resistance to flow through the pulmonary arteries that results initially from obstruction of pulmonary arterial vessels (from main to subsegmental levels) by organized thromboembolic material and subsequently from vascular remodeling in small unobstructed vessels [5].The most common complaint in CTEPH is progressive dyspnea, exercise intolerance and fatigue. Patients with CTEPH usually have a harmonious period after acute PE, during which symptoms are absent despite the onset of pulmonary hypertension [4].Initial findings of CTEPH on physical examination may include a reduction in the splitting of the second heart sound (S_2_), accentuation of the sound of pulmonic closure (P_2_), and a palpable right ventricular heave. Subsequent findings correspond to decreasing right ventricular function, jugular venous distention, fixed splitting of S_2_, a right-sided third heart sound (S_3_), tricuspid regurgitation, hepatomegaly, ascites, and peripheral edema [4]. TTE can provide the first clue of CTEPH. It can confirm elevated pulmonary pressures and show right-heart chamber enlargement, abnormal right ventricular systolic function, paradoxical interventricularseptal motion, and the impact of an enlarged right ventricle on left ventricular filling [6]. In rare cases, TTE may directly show proximal pulmonaryartery thrombus, as in our case [7]. CTEPH is usually diagnosed on the basis of radiological investigations. Ventilation-perfusion lung scanning may be used to differentiate CTEPH from other causes of pulmonary hypertension [8].Chest CT is used widely for diagnosis of CTEPH. It can identifyeccentric thromboembolic material in the pulmonary arteries,especially in the main and lobar pulmonary arteries [9].In patients with CTEPH, pulmonarythromboendarterectomy is the treatment of choice. It can remove obstructive thromboembolic material and markedly improves the hemodynamic measure of mean pulmonaryartery pressure, pulmonary vascular resistance, and cardiac output [10, 11].Drugs such as bosentan, prostacyclinand iloprostmay be used in CTEPH, but warfarinmustbe taken life-long as definitive treatment. The prognosis without treatment is poor in cases of CTEPH, and progressive pulmonary hypertension, right ventricular failure, and ultimately death are the expected outcomes [12]. When the initial mean pulmonary pressure is above 50mmHg, the 10-year survival rate is only 5% [13].

In conclusion, CTEPH is one of the important causes of pulmonary hypertension. It should be specifically considered in the diagnostic workup of patients with pulmonary hypertension. TTE and chest CT is useful for the diagnosis and follow-up of CTEPH.
